# Short-Term L-Citrulline Supplementation Does Not Affect Inspiratory Muscle Oxygenation and Respiratory Performance in Older Adults

**DOI:** 10.3390/nu15081951

**Published:** 2023-04-18

**Authors:** Anastasios A. Theodorou, Panagiotis N. Chatzinikolaou, Nikos V. Margaritelis, Filippos Christodoulou, Themistoklis Tsatalas, Vassilis Paschalis

**Affiliations:** 1 Department of Life Sciences, School of Sciences, European University Cyprus, 1516 Nicosia, Cyprus; f.christodoulou@external.euc.ac.cy (F.C.); ttsatalas@uth.gr (T.T.); 2 Department of Physical Education and Sport Science at Serres, Aristotle University of Thessaloniki, 61122 Thessaloniki, Greece; chatzinpn@phed-sr.auth.gr (P.N.C.); nvmargar@auth.gr (N.V.M.); 3 Department of Physical Education & Sport Science, University of Thessaly, 42100 Trikala, Greece; 4 School of Physical Education and Sport Science, National and Kapodistrian University of Athens, 17237 Athens, Greece

**Keywords:** nitric oxide, ergogenic aids, sports nutrition, respiratory muscles, pulmonary function, fatigue, blood flow

## Abstract

In sports nutrition, nitric oxide (NO**^•^**) precursors such as L-citrulline are widely used to enhance NO**^•^** bioavailability, which is considered an ergogenic aid. Our study aimed to examine the effect of short-term L-citrulline supplementation on respiratory muscles’ performance, fatigue, and oxygenation in older adults. Fourteen healthy older males took 6 g of L-citrulline or a placebo for seven days in a double-blind crossover design. Pulmonary function via spirometry (i.e., forced expired volume in 1 s (FEV_1_), forced vital capacity (FVC), and their ratio)), fractional exhaled nitric oxide (NO^•^), maximal inspiratory pressure (MIP), rate of perceived exertion, and sternocleidomastoid muscle oxygenation (i.e., oxyhemoglobin (Δ[O_2_Hb]) and de-oxyhemoglobin (Δ[HHb]), total hemoglobin concentration (Δ[tHb]), and tissue saturation index (TSI%)) were evaluated at baseline, after seven days of L-citrulline supplementation, and after incremental resistive breathing to task failure of the respiratory muscles. The exhaled NO**^•^** value was only significantly increased after the supplementation (26% *p* < 0.001) in the L-citrulline condition. Pulmonary function, MIP, rate of perceived exertion, and sternocleidomastoid muscle oxygenation were not affected by the L-citrulline supplementation. In the present study, although short-term L-citrulline supplementation increased exhaled NO**^•^**, no ergogenic aids were found on the examined parameters at rest and after resistive breathing to task failure in older adults.

## 1. Introduction

Nitric oxide (NO**^•^**) is a simple gaseous molecule in the human body and a free radical produced by NO^•^ synthase enzymes with a plethora of diverse biological functions [1]. In skeletal muscle, NO**^•^** has been recognized as a critical signaling molecule for muscle function, metabolism, and redox status [2,3,4,5]. The importance of NO**^•^** for muscle function and metabolism is emphasized by the fact that the major isoforms of NO^•^ synthase enzymes are found in muscle cells [3], and their activity increases several times in a contracting muscle [6]. The most common characteristic of NO^•^ is its vasodilatory function in controlling microvascular blood flow and muscle oxygenation [7,8,9]. In addition, NO**^•^** appears to regulate muscle mitochondrial oxygen consumption [10,11], force production [2,12], myocyte differentiation and muscle stem cell activation [13,14,15], and glucose metabolism [16,17].

In sports nutrition, the precursors of NO**^•^**, such as L-citrulline and L-arginine, are widely used to enhance NO**^•^** synthesis [18,19]. However, the scientific findings about the effectiveness of L-citrulline and L-arginine in improving exercise performance remain controversial [20,21,22,23]. Yet, studies employing L-citrulline supplementation are more consistent regarding their ergogenic effect on exercise performance [24,25,26,27,28,29,30]. L-citrulline’s dominance over L-arginine is attributed to its ability to bypass hepatic metabolism and then, in the kidneys, is catabolized to L-arginine [31,32]. On the other hand, during L-arginine ingestion, hepatocytes withdraw L-arginine from portal blood; hence, NO^•^ synthesis is hampered [33]. Therefore, L-citrulline administration is a more efficient precursor of L-arginine than L-arginine itself [34].

L-citrulline is a non-proteinogenic alpha-amino acid [31] essential in human health and function [35,36]. The de novo formation and release of L-citrulline in the bloodstream occur in the small intestinal enterocytes from amino acids (e.g., glutamine) derived from the diet or systemic circulation [31,37]. Yet, the de novo synthesis of L-citrulline is considered minor [38], and the conversion of L-arginine to NO^•^ in a reaction catalyzed by NO^•^ synthase enzymes in the NO^•^ cycle is the primary source of L-citrulline synthesis [31,37]. Moreover, L-citrulline participates in the urea cycle in the liver as an intermediate substance [31]. Though the synthesis of L-citrulline in the liver is compartmentalized, there is no release of L-citrulline in circulation, nor can hepatocytes uptake L-citrulline from circulation [32,37]. The food sources of L-citrulline are limited, and the primary dietary intake comes from watermelons, where this amino acid is in high concentrations [39,40]. Thus, obtaining the appropriate amount of L-citrulline from the diet is difficult. As a result, exogenous supplementation might be the most practical way to take sufficient amounts of L-citrulline to enhance L-arginine levels and NO^•^ synthesis. 

Low levels of NO^•^ have been reported to impair microvascular blood flow, vasodilation, and muscle performance [3,20,41], which is common in older or clinical populations. Indeed, older adults are characterized by decreased NO^•^ bioactivity [42,43] and have been shown to exhibit hampered blood flow [44,45], endothelial dysfunction, and oxidative capacity [46,47], as well as decreased exercise performance, muscle function, and mass [48,49]. Many studies have used L-citrulline to increase NO^•^ bioavailability and reverse microvascular and muscle function impairments. Indeed, L-citrulline supplementation increases muscle oxygenation and oxygen uptake in young men [25] and microvascular blood flow during exercise in older men [27]. These findings are promising since enhancing blood flow and oxygen supply to the working muscles is vital during exercise as the demand of the contracting muscles for oxygen and the removal of metabolic by-products is increasing [50,51].

Considering the importance of inspiratory muscles on locomotor muscle performance [52,53], we wanted to examine the impact of short-term L-citrulline supplementation on respiratory muscles’ performance, fatigue, and oxygenation in older adults. In a previous study by our group, acute supplementation with 6 g of L-citrulline in young individuals was found to increase NO^•^ bioavailability but not the respiratory performance and inspiratory muscle oxygenation measured on sternocleidomastoid muscles [54]. Therefore, we hypothesized that a more extended supplementation period might induce the ergogenic aids we failed to find after an acute dose of L-citrulline [23]. We decided to examine the sternocleidomastoid muscle as it contributes to pressure generation in strenuous inspiratory efforts [55,56], such as the resistive breathing to task failure protocol used in the present study. We believe that one is more likely to find an effect of a chronic short-term nutritional intervention (i.e., L-citrulline in this study) on metabolism and performance when a stimulus (i.e., resistive breathing) has disturbed homeostasis. Hence, the present investigation’s purpose was to examine if short-term L-citrulline supplementation can enhance NO^•^ bioavailability and cause ergogenic aids on respiratory performance, fatigue, and muscle oxygen metabolism at rest and after the incremental resistive breathing to task failure protocol.

## 2. Materials and Methods

### 2.1. Participants

Fourteen healthy older males were recruited from the public via local advertisements and social media to participate in the study voluntarily (Table 1). Their body mass was assessed with the participants wearing light clothes and barefoot (Stadiometer 208, Seca, Hamburg, Germany). Participants’ height was assessed to the nearest centimeter (Stadiometer 208, Seca, UK). Their body fat percentage was determined using the Siri skinfold equation via the seven skinfolds measures equation with a Harpenden caliper (John Bull, St. Albans, UK). The participants were asked to recall if they had participated in any intense resistance or aerobic training fifteen days before their inclusion in the experiment. The participants were not smokers and did not have pulmonary disease or other diseases that could have impaired them from intense resistive breathing. During their involvement in the study, the consumption of alcohol and caffeine was not allowed two days prior to the evaluations. The volunteers were not taking any medicines or nutritional supplements that could have influenced the evaluated parameters of the experiment. In the study, researchers asked the participants to record their food intake for two days before the first resistive breathing to task failure test. For this purpose, the researchers trained the participants with comprehensive instructions on recording their food intake and motivated them to complete this task. Prior to the second resistive breathing to task failure test, the participants were instructed to use that record and maintain identical food intakes for the two days before the evaluations. After informing the participants of all the study procedures, risks, discomforts, and benefits, the researchers obtained their written consent to participate.

### 2.2. Study Design

**Figure 1:**
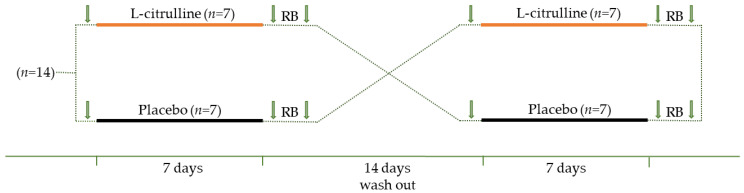
Study design. Arrows indicate the data collection time points (i.e., baseline, post-supplementation, and post-resistive breathing to task failure). RB, resistive breathing.

Our study design was counterbalanced crossover, double-blind, and placebo-controlled (Figure 1). Anthropometrical data were collected at the first visit, during which, the participants were familiarized with the equipment and the measurements. In the following days after the familiarization, the volunteers returned for baseline evaluations. Baseline evaluations contained their respiratory function, exhaled NO^•^, maximal inspiratory pressure (MIP), and sternocleidomastoid muscle oxygenation. Then, the participants were instructed to take 6 g (3 g every 12 h) of L-citrulline (Now, L-citrulline Pure Powder, Bloomingdale, IL) or a placebo (maltodextrin) daily for seven days, starting from the next day. The researchers weighed the supplements with a precision balance (AES/AEJ, Kern & Sohn, Baden-Württemberg, Germany). After that, a third investigator used randomization software to randomize the volunteers so that the researchers and the volunteers did not know their group allocation. Supplements (i.e., L-citrulline and placebo) were in liquid form in identical bottles. On day 8, seven days after the supplementation, the volunteers come back to the laboratory and followed the exact measurements as before the supplementation. To eliminate acute effects, the volunteers performed all tests 12 to 14 h after consuming the last supplement the night before [57]. Then, the volunteers underwent the incremental resistive breathing to task failure protocol using a portable spirometer (K5, Powerbreath, Southam, UK) as previously described [54]. Briefly, the test included continuous inspiration in three different resistances (30 inspirations at 70%, 80%, and 90% of the MIP) until exhaustion was achieved in the last resistance stage. It was considered that task failure was achieved when the participants could not overcome the device’s resistance. Immediately after the protocols’ completion, the same evaluations were performed as before the resistive breathing. Then, the participants underwent a two-week wash-out period and repeated the same procedures and measurements in a different condition (i.e., those who took the L-citrulline took the placebo and vice versa).

### 2.3. Maximal Inspiratory Pressure and Perceived Exertion

The portable spirometer (K5, Powerbreath, Southam, UK) was used to measure MIP. Participants had to inhale through this device while the resistance gradually increased until they could not overcome it. The MIP on the final successful attempt was recorded. For the evaluation of the perceived exertion and fatigue after the resistive breathing test, the Borg scaling was used [58]. Furthermore, the number of breaths in the last stage of resistive breathing (i.e., the resistance was 90% of MIP) was considered a physiological indicator of inspiratory muscle fatigue. 

### 2.4. Pulmonary Function

Pulmonary function was assessed by the researchers evaluating forced vital capacity (FVC), forced expiratory volume in 1 s (FEV_1_), and the FEV_1_/FVC ratio with a calibrated spirometer (Cosmed Micro Quark, Rome, Italy). The volunteers took five regular breaths in the seated position before having a maximal deep inspiration followed by a maximal effort expiration for 6 s. The best score out of three attempts was recorded.

### 2.5. Fractional Exhaled NO^•^

To evaluate fractional exhale NO^•^, the volunteers had to breathe out an automatic device (NIOX VERO, Circassia, Oxford, UK). During the attempt, the airflow, as well as the exhaled air volume, was monitored by the device for six seconds.

### 2.6. Near-Infrared Spectroscopy Measurement

For the non-invasive evaluation of sternocleidomastoid muscle microvascular concentrations of oxyhemoglobin (Δ[O_2_Hb]), de-oxyhemoglobin (Δ[HHb]), and the tissue saturation index (TSI%), during the resistive breathing to task failure, Νear-Infrared Spectroscopy was used (NIRS) (PortaMon, Artinis Medical Systems, Elst, Netherlands), as described in a previous study employing the same protocol [54]. The researchers calculated the total hemoglobin concentration (Δ[tHb]) as the sum of O_2_Hb and HHb. The muscle oxygenation parameters were assessed at baseline, during the three resistive breathing stages intensities, and after the end of the protocol (i.e., 5 min recovery).

### 2.7. Statistical Analysis

The Sharipo-Wilk test was used to examine the distribution of all dependent variables, which was normal. For MIP, NO^•^, rate of perceived exertion, FVC, FEV_1_, and FEV_1_/FVC, a two-way repeated-measures ANOVA test ((group (L-citrulline vs. placebo) × time (baseline, pre-resistive breathing, and post-resistive breathing)) was followed. A second two-way repeated-measures ANOVA test was performed for Δ[O_2_Hb], Δ[HHb], Δ[tHb], and TSI% ((group (L-citrulline vs. placebo) × time (baseline, pre-resistive breathing, 70%, 80%, and 90% exercise intensity and recovery)). A paired t-test was performed for the number of breaths in the last stage of resistive breathing. If a significant interaction was found, pairwise comparisons were performed using the Sidak test. Data are given as mean  ±  standard deviation (SD), and the level of significance was set at a = 0.05. For all the statistical analyses, the researchers used the ΙΒΜ SPSS version 25.0 software. 

## 3. Results

### 3.1. Fractional Exhaled NO^•^

**Figure 2:**
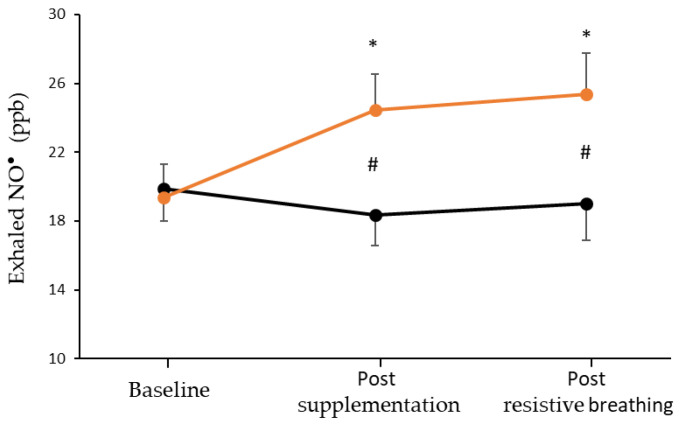
Exhaled NO^•^ at baseline, after seven days of L-citrulline (orange circles) and placebo (black circles) supplementation and post resistive breathing to task failure. (*) indicates significant difference (*p* < 0.001) compared to baseline; (#) indicates significant difference (*p* < 0.05) between the L-citrulline and placebo conditions.

A significant condition by time interaction (*p* < 0.001) and a main effect of time (*p* = 0.006) was found in the exhaled NO^•^ concentration, but no main effect of condition was found (*p* = 0.126) (Figure 2). Specifically, after seven days of L-citrulline supplementation, the exhaled NO^•^ concentration increased (+5.29 ppb; 95% CI [2.55, 8.02], *p* < 0.001), and this increase remained until the end of the incremental resistive breathing to task failure (+6.36 ppb; 95% CI [3.32, 9.39], *p* < 0.001). On the contrary, no difference was observed in exhaled NO^•^ for the placebo condition post-supplementation (−1.50 ppb; 95% CI [−4.24, 1.24], *p* = 0.436) or at the end of the resistive breathing protocol (−0.86 ppb; 95% CI [−3.89, 2.18], *p* = 0.857). The differences between the two conditions were equal to 6.29 ppb (95% CI [0.48, 12.09], *p* = 0.035) and 6.71 ppb (95% CI [0.75, 12.68], *p* = 0.029) post-supplementation and at the end of the resistive breathing protocol, respectively. 

### 3.2. Inspiratory Muscles Performance, Perceived Exertion, and Fatigue

**Figure 3:**
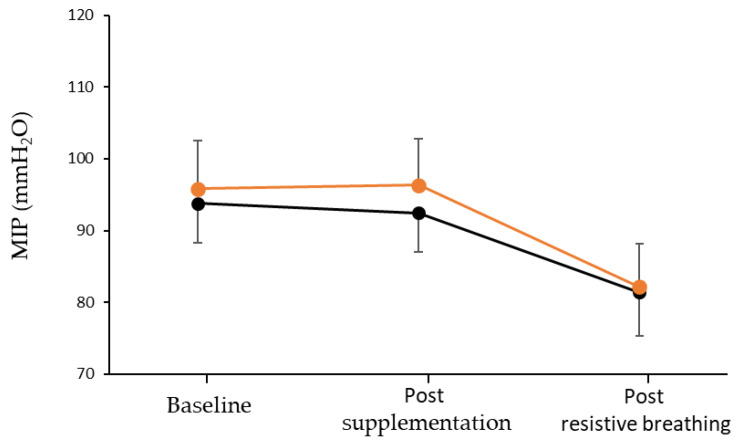
Maximal inspiratory pressure (MIP) at baseline, after seven days of L-citrulline (orange circles) and placebo (black circles) supplementation and post resistive breathing to task failure.

Concerning MIP, no significant condition by time interaction (*p* = 0.669) or main effect of condition (*p* = 0.785) was reported. However, a significant main effect of time was found (*p* < 0.001; Figure 3). More specifically, both conditions exhibited similar declines in MIP during the incremental resistive breathing to task failure compared to post-supplementation (L-citrulline condition: −14.21 mmHg; 95% CI [−23.25, −5.18]; and placebo condition: −11.00 mmHg; 95% CI [−20.04, −1.96]). 

Regarding the Borg scale of perceived exertion, no significant condition by time interaction (*p* = 0.548) or main effect of condition (*p* = 0.518) was reported. However, a significant main effect of time was found (*p* < 0.001; data not presented). More specifically, both conditions exhibited a similar increase in perceived exertion during the incremental resistive breathing to task failure compared to baseline (i.e., 17.9 ± 1.4 and 17.5 ± 1.5 for L-citrulline and placebo, respectively). Furthermore, during the last stage of the incremental resistive breathing (i.e., 90% of the maximal MIP), the number of breaths at task failure was not different between the L-citrulline (38.4 ± 14.1 breaths) and the placebo (36.1 ± 13.9 breaths) condition (+2.3 breaths; 95% CI [−7.86, 3.29], *p* = 0.391; data not presented). 

### 3.3. Respiratory Muscles Capacity and Sternocleidomastoid Muscle Oxygenation

**Figure 4:**
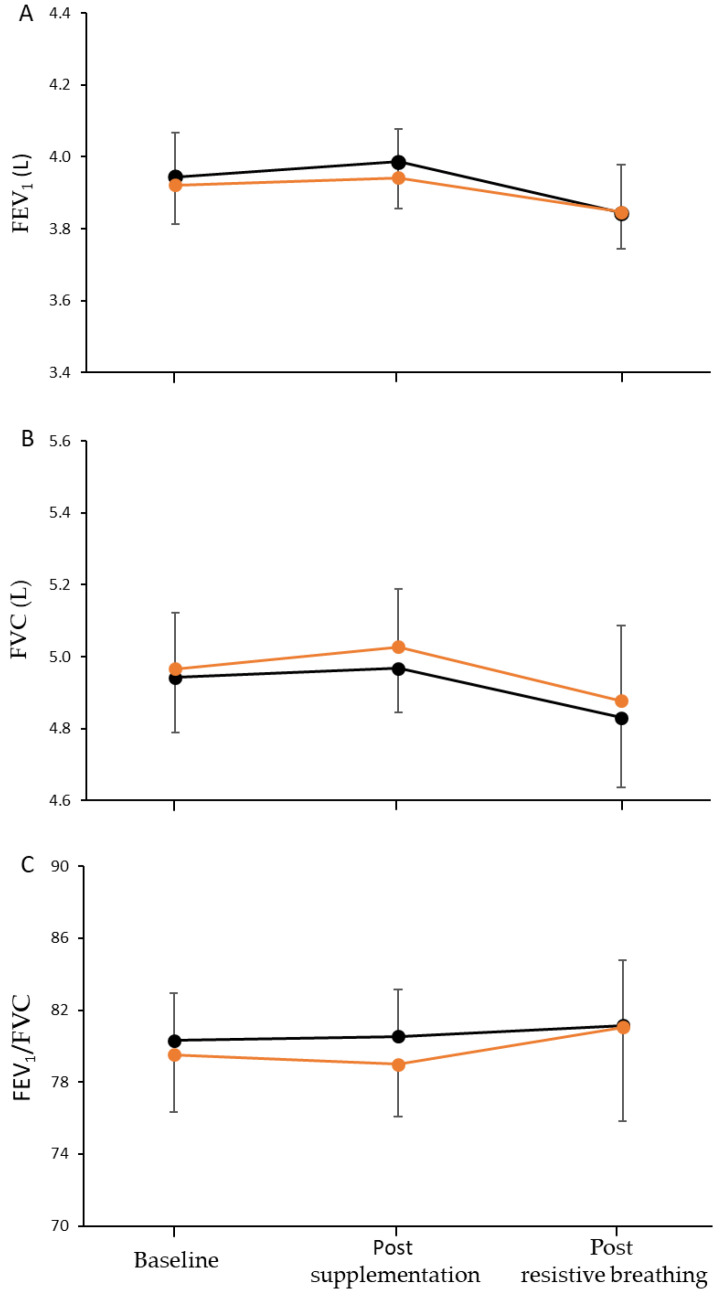
Forced expired volume in 1 second (**A**), forced vital capacity (**B**), and their ratio (**C**) at baseline, after seven days of L-citrulline (orange circles) and placebo (black circles) supplementation and post resistive breathing to task failure.

No significant main effect of time, condition or condition-by-time interaction was found in FEV_1_ (*p* = 0.124, *p* = 0.900, and *p* = 0.919, respectively), FVC (*p* = 0.333, *p* = 0.845, and *p* = 0.913, respectively), and FEV_1_/FVC (*p* = 0.878, *p* = 0.850, and *p* = 0.940, respectively) (Figure 4). Thus, FEV1, FVC, and their ratio were not affected by the L-citrulline supplementation or by the respiratory muscles’ resistive breathing to task failure test.

**Figure 5:**
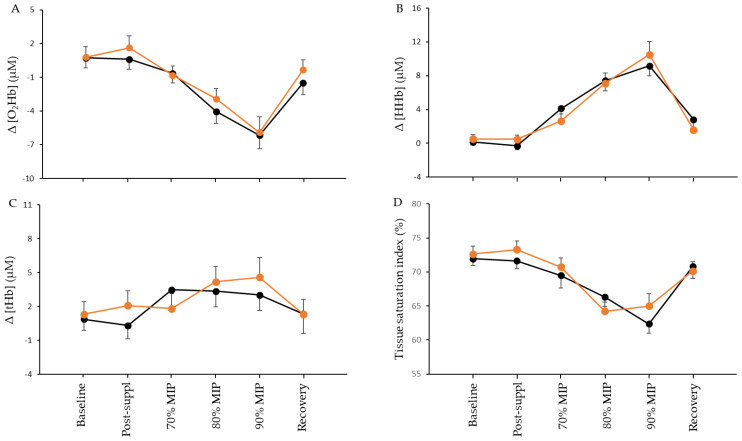
Sternocleidomastoid muscle oxyhemoglobin (O_2_Hb) (**A**), deoxyhemoglobin (HHb) (**B**), total hemoglobin (tHb) (**C**), and tissue saturation index (**D**) at baseline, after seven days of L-citrulline (orange circles) and placebo (black circles) supplementation and post resistive breathing to task failure.

Concerning muscle oxygenation, neither a main effect of condition nor a condition by time interaction was found in Δ[O_2_Hb] (*p* = 0.591 and *p* = 0.815, respectively), Δ[HHb] (*p* = 0.902 and *p* = 0.653, respectively), Δ[tHb] (*p* = 0.684 and *p* = 0.708, respectively), and TSI% (*p* = 0.782 and *p* = 0.524, respectively) between L-citrulline and the placebo measured in sternocleidomastoid muscles (Figure 5A–D). Nevertheless, a significant main effect of time was found for Δ[O_2_Hb] (*p* < 0.001), Δ[HHb] (*p* < 0.001), and TSI% (*p* < 0.001). More specifically, the resistive breathing intensity stages of 80% and 90% MIP caused a Δ[O_2_Hb] decrease in both the L-citrulline (at 80%: −3.71 μΜ; 95% CI [−7.11, −0.31,] and at 90%: −6.69 μΜ; 95% CI [−10.72, −2.68]) and placebo (at 80%: −4.78 μΜ; 95% CI [−8.18, −1.37] and at 90%: −6.89 μΜ; 95% CI [−10.91, −2.87]) conditions. Likewise, the breathing intensity stages of 80% and 90% MIP caused a TSI% decrease in both the L-citrulline (at 80%: −8.4%; 95% CI [−13.67, −3.18] and at 90%: −7.64%; 95% CI [−13.74, −1.55]) and placebo (at 80%: −5.64%; 95% CI [−10.89, −0.39] and at 90%: −9.57%; 95% CI [−15.67, −3.48]) conditions. Finally, a Δ[HHb] increase was observed compared to baseline in the 80% and 90% MIP stages in both the L-citrulline (at 80%: +6.60 μΜ; 95% CI [2.03, 11.18] and at 90%: +9.99μΜ; 95% CI [5.13, 14.84]) and placebo (at 80%: +7.27 μΜ; 95% CI [2.69, 11.84] and at 90%: +9.03 μΜ; 95% CI [4.18, 13.89]) conditions. After recovery, all muscle oxygenation parameters returned to the baseline values.

## 4. Discussion

To our knowledge, this is the first study investigating the effect of short-term L-citrulline supplementation on respiratory muscles’ performance, fatigue, and oxygenation in older persons. We hypothesized that L-citrulline supplementation for seven days would increase NO^•^ bioavailability, thereby improving respiratory muscles’ performance, resistance to fatigue, and sternocleidomastoid muscle oxygenation at rest and during incremental resistive breathing to task failure. Contrary to our hypothesis, although short-term L-citrulline supplementation significantly increased exhaled NO^•^, no ergogenic effects were observed in the examined parameters. 

In a previous investigation, we reported no positive effect of acute L-citrulline supplementation on sternocleidomastoid muscle oxygenation and respiratory performance in healthy young individuals [54]. This could be attributed to the acute supplementation dose, and it is plausible that a more extended supplementation period may induce greater effects. Indeed, a recent systematic review paper suggested that chronic L-citrulline supplementation might be more effective in positively affecting performance than acute supplementation [23]. Thus, we decided to proceed with a 7-day supplementation period in the present investigation. In addition, we recruited older persons, as this age group is characterized by reduced muscle blood flow [44,45], endothelial dysfunction [42], and decreased NO^•^ bioactivity [43]. In our opinion, we were more likely to find an effect of L-citrulline when the intervention was targeted [59,60] to a population characterized by impairments on the examined parameters. 

### 4.1. L-Citrulline Supplementation and NO^•^ Bioavailability

In sports nutrition, L-citrulline supplementation aims to boost extracellular L-arginine, thus enhancing NO**^•^** synthesis and bioavailability since L-arginine synthesizes NO**^•^** in the endothelium. L-citrulline has the advantage of bypassing intestinal and vascular arginase activity (which contests with NO**^•^** synthases for L-arginine), hepatic metabolism, and blood withdrawal [32,37,61]. In our study, 6 g of L-citrulline taken twice daily for seven days increased exhaled NO**^•^** by 26%. It is worth noting that this supplementation dose and duration are considered safe and well-tolerated [57]. In our previous study, in which 6 g of L-citrulline was taken in a single dose one hour before, we observed an increase of 19% in exhaled NO**^•^** [54]. Thus, it appears that both an acute and a short-term supplementation protocol of L-citrulline are effective in increasing exhaled NO**^•^**. However, in our study, the absence of NO**^•^** production assessments in skeletal muscle and plasma is a considerable limitation that restricted us from meaningful information, taking into account the different origins of NO^•^ in skeletal muscle [62] and blood [63]. Other studies following similar dose and duration protocols reported controversial results [64,65]. More specifically, Ochai et al. [65] reported that 5.6 g of L-citrulline for seven days increased plasma NO**^•^** concentration in middle-aged men. On the contrary, Essen et al. [66] reported no effect after the supplementation of 8 g of L-citrulline for eight days in young male and female athletes. It is clear that additional studies providing mechanistic information regarding NO**^•^** production and metabolism after L-citrulline supplementation are required to extract solid conclusions. Therefore, we suggest that future studies include the measurement of NO**^•^** of different origins and compartments for a more holistic approach. 

### 4.2. Inspiratory Muscle Performance and Resistance to Fatigue

Fatigue is the most common sensation everyone can physiologically experience during physical activity and the main exercise-limiting factor [67,68]. It may arise from different molecular and biochemical processes and mechanisms, all contributing to a significant decline in performance [67,68]. Inspiratory muscles are prone to fatigue, negatively affecting locomotor muscle performance and exercise capacity [53,69]. Increased work of breathing triggers a respiratory muscle metaboreflex enhancing sympathetic nerve activity and vasoconstrictors in the locomotor muscles, reducing blood flow at rest [70] and during exercise [53]. 

In the present investigation, we assumed that increasing inspiratory muscle blood flow and oxygen delivery via short-term L-citrulline supplementation would positively affect respiratory muscle performance and resistance to fatigue. To investigate this hypothesis, we examined respiratory muscle performance at rest and after a fatiguing incremental resisting breathing protocol to task failure. As expected, resistive breathing to task failure was effective in causing a drop in the participants’ respiratory performance and severe fatigue. The MIP was significantly lower after the breathing test. Moreover, the perceived exertion score was significantly higher, and the number of breaths at exhaustion during the last stage was significantly lower. However, even though NO**^•^** was significantly increased in the L-citrulline condition, the supplement failed to improve inspiratory muscle performance at rest and post a resisting breathing to task failure test. Likewise, the short-term L-citrulline supplementation did not alter FEV1, FVC, and the ratio between them. Similar results were found after acute L-citrulline supplementation in young males using the same resisting breathing test [54]. Thus, it could be suggested that acute and short-term L-citrulline supplementation does not have ergogenic aids on muscle performance or resistance to fatigue. Undoubtedly, further investigations are needed on the effect of L-citrulline on respiratory muscle performance, as our studies are the only ones that have examined this scenario. Recent studies using comparable supplementation doses and durations reported contradictory results on exercise performance. Specifically, other studies found that L-citrulline has positive effects on the rate of perceived exertion during cycling [29] and cycling performance [25,29,30] but has no impact on swimming performance [64] and the rate of perceived exertion after cycling [30]. It is worth noting that the above studies were performed on active young individuals, whereas the present investigation was on older adults. A study on older adults [26] reported increased gait speed after L-citrulline supplementation. However, compared to the current investigation, Buckinx et al. [26] used a higher dose of L-citrulline (10 g), which was supplemented for a longer time (12 weeks). Additionally, the supplementation was combined with high-intensity interval training.

### 4.3. Sternocleidomastoid Muscle Oxygenation and Blood Flow

NIRS technology was employed in our study to examine the effect of L-citrulline supplementation on sternocleidomastoid muscle oxygenation at rest and during incremental resistive breathing to task failure. We chose the sternocleidomastoid muscle since it is highly active during exercise [66] and during incremental inspiratory loading [71], which was the case in our resistive breathing to task failure. As expected, during the resistive breathing, significant decreases in Δ[O_2_Hb] and TSI% were observed. In contrast, increases in Δ[HHb] were found probably to facilitate oxygen supply to the working sternocleidomastoid muscle. These findings align with our results after acute L-citrulline supplementation [54] and work from others [71]. Additionally, they confirm that the adopted incremental resistive breathing protocol could induce hemodynamic changes in the examined muscle.

Nevertheless, despite the increase in exhaled NO**^•^** after the supplementation and considering the role of NO**^•^** on blood flow, in this examination, 6 g of L-citrulline for seven days could not induce any changes in sternocleidomastoid muscle oxygenation. Contrary to our results, Bailey et al. [25] reported improvements in VO_2_ kinetics and the (HHb) amplitude of *vastus lateralis* during moderate cycling after supplementation with 6 g/d of L-citrulline for seven days in young males. In addition, Gonzales et al. [27] found enhanced femoral blood flow by 11% during submaximal exercise in older males after supplementation with 6 g/d of L-citrulline for 14 days. However, in that investigation, L-citrulline did not improve blood flow in older females.

### 4.4. NO^•^ Precursors and Vascular Function

NO^•^ is a potent vasodilator that controls vascular endothelial function and blood flow [72,73]. It controls microvascular function by activating the soluble guanylate cyclase pathway in the vascular smooth muscle cells (Simmonds et al., 2014; Tejero et al., 2019). The soluble guanylate cyclase converts guanosine triphosphate to cyclic guanosine monophosphate, leading to decreased sarcoplasmic Ca^2+^ levels and, thereby, resulting in smooth muscle relaxation, vascular wall expansion, and increased blood flow. Thus, NO^•^ precursors might favorably affect vascular function by increasing NO^•^ synthesis and bioavailability [21,74]. Certainly, apart from NO^•^, several other molecules and pathways could influence vascular function and blood flow. Studies suggest that L-citrulline and L-arginine may reduce blood pressure and arterial stiffness [75,76,77,78]. For L-arginine, a meta-analysis of randomized placebo-controlled trials showed that L-arginine supplementation between 4 g to 24 g per day significantly reduced systolic and diastolic blood pressure [79]. Another recent meta-analysis found that long-term L-citrulline supplementation significantly improved brachial-artery-flow-mediated vasodilation, the gold-standard non-invasive tool for evaluating endothelial function [80]. The ambiguous reports on the role of L-citrulline and L-arginine in vascular function may be due to the different supplementation schemes (i.e., dose and duration), experimental protocols, and different populations (e.g., healthy individuals or with heart failure).

### 4.5. Conclusions

In the present study, 6 g of L-citrulline supplementation for seven days significantly increased NO^•^ bioavailability in older males. A key element in the present study was enrolling older adults who could benefit from L-citrulline supplementation, as this group is characterized by impaired NO^•^ bioactivity and hampered blood flow. However, despite the theoretically favorable role of NO^•^ on exercise performance [2,4,20], no ergogenic effects of L-citrulline supplementation were observed in respiratory muscles’ performance, fatigue resistance, and sternocleidomastoid oxygen metabolism. Previously, we found similar results after acute L-citrulline supplementation in young males. Therefore, although studies report ergogenic effects of L-citrulline on vascular function and exercise performance [24,25,26,27,28,29,30,76,77], our findings show that L-citrulline supplementation might not have implications for respiratory muscles performance enhancement. The lack of significant findings on respiratory muscle oxygenation and performance could be attributed to the fact that the potential systemic effects of L-citrulline were difficult to detect in a single muscle such as the sternocleidomastoid. Furthermore, several molecules and processes have been reported to interact to fine-tune microvascular function in different conditions [73], such as during high and low oxygen availability [5]. Thus, it is challenging to gain mechanistic insights by only measuring a single molecule of a complex pathway controlling vascular function. Based on this, future studies on the role of L-citrulline should measure NO^•^ production in different cellular compartments (e.g., blood and muscle), monitor muscle oxygenation and blood flow in multiple muscle groups which are active during exercise, and implement whole-body exercise protocols that depend highly on aerobic metabolism.

## Figures and Tables

**Table 1:** Anthropometric characteristics of the participants (mean ± SD).

	*n* = 14		
Age (yr)	63.1	±	5.5
Height (cm)	174	±	3.9
Weight (kg)	78.8	±	6.8
BMI (kg/m^2^)	26.1	±	2.5
Body fat (%)	26.4	±	2.6
Waist circumference (cm)	98.4	±	5.5
Hip circumference (cm)	103.7	±	6.6
Waist-to-hip ratio	0.95	±	0.02

## Data Availability

Data from the current study are available from the corresponding author upon reasonable request.
